# C-reactive protein-to-albumin ratio and neutrophil-to-albumin ratio for predicting response and prognosis to infliximab in ulcerative colitis

**DOI:** 10.3389/fmed.2024.1349070

**Published:** 2024-03-12

**Authors:** Yanyan Zhang, Feng Xu, Ya Li, Bing Chen

**Affiliations:** Department of Gastroenterology, The First Affiliated Hospital of Zhengzhou University, Zhengzhou University, Zhengzhou, China

**Keywords:** ulcerative colitis, inflammatory bowel disease, biomarker, C-reactive protein-to-albumin ratio, neutrophil-to-albumin ratio, drug efficacy

## Abstract

The C-reactive protein-to-albumin ratio (CAR) and neutrophil-to-albumin ratio (NAR) serve as established markers for inflammatory diseases. However, limited studies have investigated their potential in predicting response and prognosis following infliximab (IFX) treatment. The objective of this paper was to evaluate feasibility of CAR and NAR as biomarkers to assess response to IFX induction therapy. Additionally, we attempted to determine the capacity to predict clinical remission in ulcerative colitis (UC) after 54 weeks of IFX treatment. We enrolled a total of 157 UC patients diagnosed via endoscopic mucosal biopsy at our hospital between October 2018 and June 2023. Additionally, 199 patients presenting with gastrointestinal symptoms, who underwent physical examinations, constituted the control group. Comprehensive clinical data, laboratory indicators, and endoscopic findings were systematically collected. CAR and NAR values were computed before treatment, post-induction, and subsequently at 8-week intervals. Comparisons between two groups were analyzed using the Wilcoxon rank-sum test or the independent samples *t*-test, and comparisons between multiple groups were analyzed using the one-way ANOVA (analysis of variance) or the Kruskal-Wallis rank sum test. We found CAR and NAR emerged as sensitive biomarkers for assessing disease activity. Notably, our findings indicated their dual predictive capability: foreseeing response post-IFX induction therapy and prognosticating the likelihood of UC patients achieving clinical remission following 54 weeks on IFX therapy.

## 1 Introduction

Ulcerative colitis (UC) is an inflammatory bowel disease (IBD) predominantly affecting the rectum or colon, with its incidence projected to rise to 5 million cases by 2023. Its typical feature is the recurrence and remission of diffuse mucosal inflammation, and the typical clinical manifestation is mucopurulent bloody stool, which is diagnosed depending on endoscopic histological findings and clinical manifestations. The complex pathophysiology comprises genetic susceptibility, epithelial barrier abnormalities, dysregulated immunological responses, and environmental factors (1). Treatment options include medical and surgical interventions, with 5-aminosalicylic acid being the preferred therapy; however, alternatives like corticosteroids, thiopurines, biological agents, or small-molecule drugs might be considered if initial therapy is ineffective. Patients with inadequate responses to drug therapy might require total rectocolectomy, which poses significant postoperative complications and increased medical burdens. Recent advancements in drug therapies have shifted treatment goals toward achieving clinical remission and prioritizing mucosal healing on histological examination. Timely and accurate assessment of disease is crucial for selecting appropriate treatment strategies, which often relies on biomarkers and endoscopic evaluations. Despite the gold standard status of endoscopy, its limitations persist due to its invasiveness, high cost, and low patient compliance (2, 3). Consequently, non-invasive, and straightforward biomarkers to assess disease activity and predict drug efficacy are urgently needed.

Recent studies have explored biomarkers for assessing disease, such as the use of fecal calprotectin in gauging IBD activity (4), which was limited by high costs and lengthy processing times. Commonly used biomarkers like C-reactive protein (CRP) and erythrocyte sedimentation rate (ESR) also face challenges due to their susceptibility to various elements, including bacterial and viral infections, compromising their sensitivity and specificity (5). At the same time, research on the assessment of drug efficacy has been increasing in recent years. The therapy of UC has significantly evolved with the widespread use of biologics (such as anti-TNF drugs). It results in clear benefits to the patients, significantly prolonging the duration of remission and improving the life quality of patients. However, efficacy is not achieved in all patients who receive treatment with biologics, and approximately 30 percent of patients experience no respond to the biological agents. Therefore, timely and effective assessment of drug efficacy is crucial to the prognosis of patients, avoiding delays in treatment and reducing the cost of treatment for patients. Single biomarkers have limitations in predicting disease progression, prompting increased research into combining multiple biomarkers, for example, NAR forecasted the reaction to IFX in Crohn’s disease (CD) patients (6), neutrophil-lymphocyte ratio (NLR) and platelet-lymphocyte ratio (PLR) were used as new biomarkers of mucosal prognosis for UC patients receiving anti-TNF therapy (7, 8).

In this paper, the objective was to investigate potential of CAR and NAR as biomarkers for evaluating treatment response post-IFX induction therapy in UC patients. Additionally, our aim was to assess whether CAR and NAR can predict the attainment of clinical remission among UC patients following a 54-week IFX treatment regimen.

## 2 Materials and methods

### 2.1 Study population

This research was authorized by the Ethics Review Board of the Clinical Research Institution of the First Affiliated Hospital of Zhengzhou University. The study comprised 157 patients hospitalized to our hospital between October 2018 and June 2023, diagnosed using endoscopic mucosal biopsy. Additionally, 199 individuals exhibiting gastrointestinal symptoms underwent physical examinations and served as controls. The inclusion standards were established as such: (1) Patients aged 18 years and above. (2) Patients meeting the indications for IFX treatment under the Beijing 2018 Beijing Consensus on UC Diagnosis and Treatment (9). (3) Patients who received their initial infliximab treatment at our institution. Exclusion criteria were set to exclude: (1) patients with liver or gallbladder diseases, a prolonged history of alcohol consumption, or the use of medications impacting liver function. (2) Individuals with conditions such as intestinal perforation, intestinal obstruction, cardiovascular or respiratory diseases, renal insufficiency, diabetes, cerebral infarction, malignant tumors, acute or chronic infections, other autoimmune diseases, or who had undergone gastrointestinal surgery or lacked relevant data. (3) Patients not concurrently using immunosuppressants, cortisol, or other drugs that might influence neutrophil levels.

### 2.2 Data collection

Comprehensive clinical data, laboratory indicators, and endoscopic findings were gathered, including gender, age, disease duration, stool frequency, extent of hematochezia, CRP, ESR, albumin (ALB), neutrophil count (NEU), and colonoscopy results. Moreover, CAR and NAR were computed. For patients diagnosed with UC, follow-up assessments included monitoring laboratory indicators, clinical observations, and physical examinations conducted prior to commencing IFX treatment at weeks 0, 2, and 6, and every 8 weeks thereafter.

### 2.3 Statistical analysis

Statistical analyses were performed by the Statistical Package for Social Sciences (SPSS) Version 26.0. Normally distributed data were reported as means with standard deviations (SD). Comparisons between groups of normal distribution data were conducted utilizing either the independent sample *t*-test or the one-way ANOVA. Median values with interquartile ranges [M (P25, P75)] were used to report non-normally distributed data, and group comparisons were done via the Wilcoxon rank sum test or the Kruskal-Wallis rank sum test. Count statistics were expressed as percentages or proportions. Categorical variable comparisons were undertaken via the Chi-square test and Fisher’s exact test. The relationship between parameters and Mayo score was examined by Spearman correlation analysis. To quantify responsiveness and clinical prognosis, the receiver operating characteristic (ROC) curve was applied, the cutoff value was set by the Youden index, and *P* < 0.05 was judged statistically significant.

## 3 Results

### 3.1 Patient demographics and laboratory tests

**FIGURE 1:**
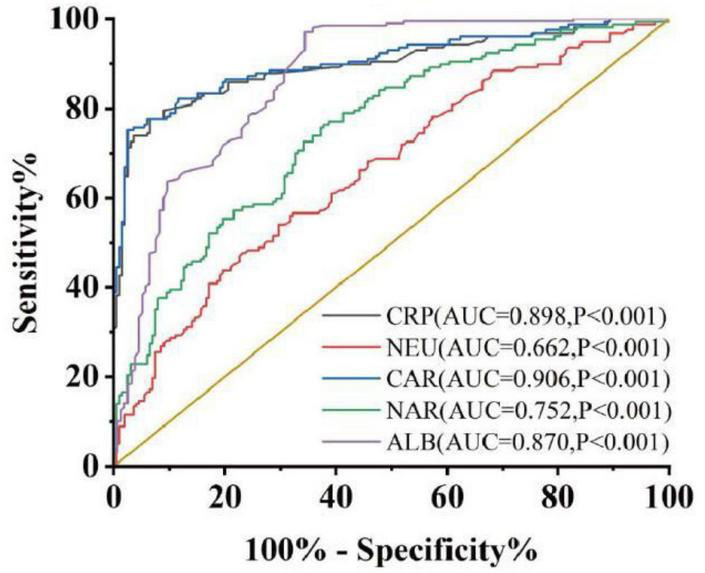
Discriminate abilities of serum parameters between case group and control group. AUC, area under the ROC curve. *P* < 0.05 was considered statistically significant.

According to the data presented in Table 1, a collective of 157 individuals diagnosed with UC was observed, comprising 71 males and 86 females. Their average age was recorded at 45.13 ± 14.84 years, and the disease duration spanned 36 (24, 96) months. Additionally, 199 individuals underwent physical examinations as controls, consisting of 88 women and 111 men; their mean age was 46.37 ± 15.53 years old. There were no notable distinctions in gender (*P* = 0.480) or age (*P* = 0.537) detected between the control and case cohorts. Similarly, no obvious difference was found in body mass index (BMI) [21.60 (19.43, 23.50) VS 21.44 (20.25, 24.03), *P* = 0.248]. Comparative analyses of whole blood cell and serum biochemical examinations between these groups revealed that NEU [4.10 (2.94, 5.99)] and CRP [10.44 (3.75, 29.12)] exhibited a marked increase in the case group compared to the control group [NEU, 3.14 (2.48, 4.24), *P* < 0.001; CRP, 1.05 (0.49, 1.77), *P* < 0.001], whereas ALB [37.10 (32.38, 40.33)] was notably lower compared to healthy controls [ALB, 43.70 (40.90, 45.80), *P* < 0.001]. These findings were consistent with previous studies (10–12). Subsequently, it was observed that both CAR [0.287 (0.100, 0.849)] and NAR [0.110 (0.082, 0.061)] were significantly increased in UC patients compared to healthy controls [CAR, 0.024 (0.011, 0.044), *P* < 0.001; NAR, 0.072 (0.055, 0.097), *P* < 0.001]. Furthermore, ROC curve assessment was undertaken to measure diagnostic accuracy (Figure 1). The larger area under the curve (AUC) indicates better diagnostic ability (13). The results revealed significant discrimination between the case and control groups for indicators (NEU, ALB, CRP, CAR, and NAR). Notably, combined indicators exhibited higher AUC values than individual ones. Specifically, NAR (the cut-off value: 0.081, sensitivity 0.764, specificity 0.623, AUC = 0.752, 95% CI 0.702–0.802, *P* < 0.001) displayed the higher AUC compared to NEU (the cut-off value: 4.29, sensitivity 0.478, specificity 0.769, AUC = 0.662, 95% CI 0.605–0.718, *P* < 0.001), while CAR (the cut-off value: 0.102, sensitivity 0.752, specificity 0.975, AUC = 0.906, 95% CI 0.872–0.939, *P* < 0.001) surpassed CRP (the cut-off value: 3.6, sensitivity 0.777, specificity 0.935, AUC = 0.898, 95% CI 0.862–0.933, *P* < 0.001) or ALB (the cut-off value: 38.55, sensitivity 0.662, specificity 0.970, AUC = 0.870, 95% CI 0.834–0.910, *P* < 0.001) alone.

**TABLE 1:** Demographics and clinical parameters in UC patients and healthy controls.

	UC patients	Healthy controls	*P*-value
Number of subjects (n)	157	199	–
Age (year)	45.13 ± 14.84	46.37 ± 15.53	0.537
BMI (Kg/m^2^)	21.60 (19.43, 23.50)	21.44 (20.25, 24.03)	0.248
Gender (n)			
Male	71 (45.2%)	111 (55.8%)	0.480
Female	86 (54.8%)	88 (44.2%)	
**Smoke**			
Yes	18 (10.9%)	26 (13.1%)	0.534
No	139 (89.1%)	173 (86.9%)	
Disease duration (months)	36.00 (24.00, 96.00)	–	–
NEU (×10^^^9/L)	4.10 (2.94, 5.99)	3.14 (2.48, 4.24)	<0.001
PLT (×10^^^9/L)	288 (227, 366)	226 (186, 275)	<0.001
ALB (g/L)	37.10 (32.38, 40.33)	43.70 (40.90, 45.80)	<0.001
CPR (mg/L)	10.44 (3.75, 29.12)	1.05 (0.49, 1.77)	<0.001
ESR (mm/h)	18.00 (9.5, 34.5)	6.00 (4.00, 9.40)	<0.001
CAR	0.287 (0.100, 0.849)	0.024 (0.011, 0.044)	<0.001
NAR	0.110 (0.082, 0.061)	0.072 (0.055, 0.097)	<0.001

Normally distributed data were presented as means ± standard deviations (SD), while non-normally distributed data were displayed as median (interquartile range) [M (P25, P75)]. Count data were represented as percentages or proportions. UC, ulcerative colitis; BMI, body mass index; NEU, neutrophil count; ESR, erythrocyte sedimentation rate; CRP, C-reactive protein; ALB, albumin; PLT, platelet; NAR, neutrophil-to-albumin ratio; CAR, C-reactive protein-to-albumin ratio. Differences among these parameters were assessed by either the independent samples *t*-test or Wilcoxon rank sum test. Gender and smoking status were examined by the Chi-square test. *P* < 0.05 which indicated the difference was statistically significant.

### 3.2 Correlation of CAR and NAR with UC activity

Subsequently, we explored whether CAR and NAR could serve as potential biomarkers to assess UC activity. The partial Mayo score was utilized as an established indicator for assessing UC activity (14). Nutritional status may influence the severity of disease, for example, Stabroth-Akil et al. (15) discovered an inverse correlation between disease severity and BMI as well (15). However, Yerushalmy-Feler et al. (16) found that children with IBD have more severe disease progression when their BMI was in the lowest or highest quartile (16), and the findings regarding the relationship between BMI and disease activity have become inconsistent at this moment. In this study, we divided the case group into moderately active and severely active groups based on the modified Mayo score, there was no significant differences in BMI between moderately active group and severely active group [BMI, 22.38 (19.27, 24.23) VS 21.14 (18.92, 23.08), *P* = 0.239], so we couldn’t yet assume that BMI correlates with UC activity. Spearman correlation analysis indicated the positive association between CAR, NAR, and Mayo scores (CAR *r* = 0.775, *P* < 0.001; NAR *r* = 0.740, *P* < 0.001) (Table 2). We further examined the correlation of Mayo scores with NEU, CRP, and ALB. These variables also exhibited positive correlations with Mayo scores. However, our findings indicated that the combination of parameters provided a more accurate assessment of disease activity compared to individual parameters. Hence, CAR and NAR might offer enhanced efficacy in evaluating UC activity compared to NEU, ALB, and CRP alone.

**TABLE 2:** Correlation between parameters and the Mayo score.

	r	*P*-value
CAR	0.775	<0.001
NAR	0.740	<0.001
CPR (mg/L)	0.765	<0.001
NEU (10^^^9/L)	0.574	<0.001
ALB (g/L)	−0.692	<0.001
ESR (mm/h)	0.731	<0.001

The link between different parameters and Mayo scores in UC patients was indicated by spearman correlation analysis. *P* < 0.05 which suggested the difference was statistically significant.

### 3.3 CAR and NAR predict response and prognosis to IFX in UC patients

**FIGURE 2:**
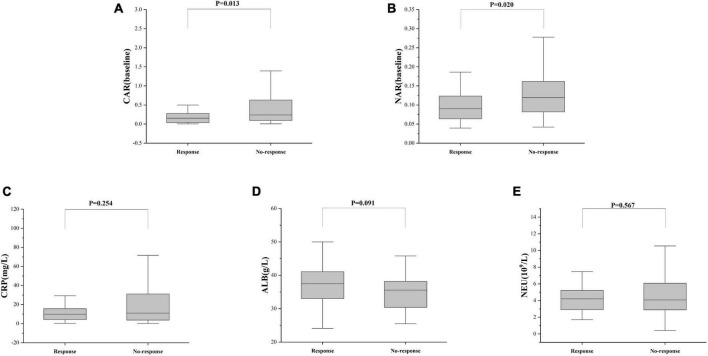
C-reactive protein and NAR predict responses after IFX induction therapy. The differences in **(A)** CAR, **(B)** NAR, **(C)** CRP, **(D)** ALB, and **(E)** NEU between responders and non-responders were determined. The comparison was performed by the Wilcoxon rank sum test [**(A)** CAR, *P* = 0.013; **(B)** NAR, *P* = 0.020; **(C)** CRP, *P* = 0.254; **(D)** ALB, *P* = 0.091; **(E)** NEU, *P* = 0.567].

**FIGURE 3:**
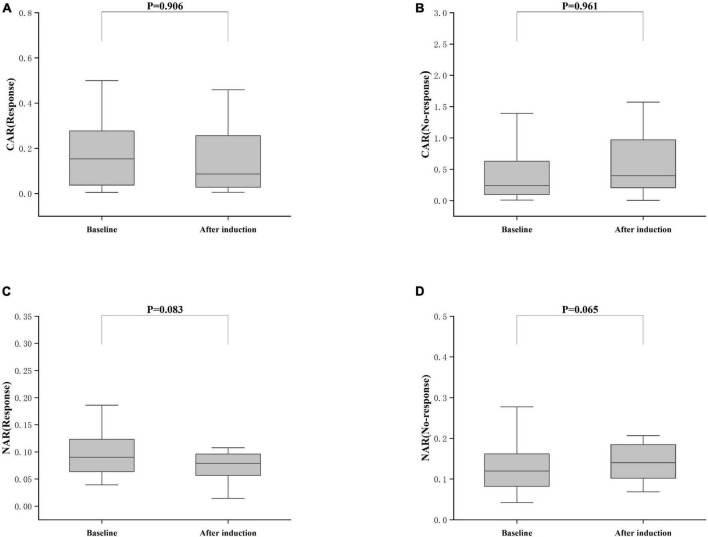
Differences between after induction CAR and NAR and baseline CAR and NAR. **(A)** CAR (Response), CAR with response after induction therapy; **(B)** CAR (No-response), CAR with no response after induction therapy; **(C)** NAR (Response), NAR with response after induction therapy; **(D)** NAR (No-response), NAR with no response after induction therapy; The comparison was performed by the Wilcoxon rank sum test [**(A)** CAR (Response), *P* = 0.906; **(C)** NAR (Response), *P* = 0.083; **(B)** CAR (No-response), *P* = 0.961; **(D)** NAR (No-response), *P* = 0.065].

**FIGURE 4:**
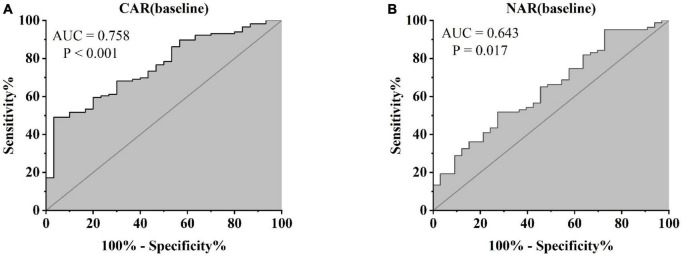
Abilities of CAR and NAR to distinguish between responders and non-responders [**(A)** CAR, AUC = 0.758, *P* < 0.001; **(B)** NAR, AUC = 0.643, *P* = 0.017, ROC curve analysis].

Treatment approaches for UC have witnessed evolution, particularly with the widespread use of biologics such as IFX. Despite early therapy escalation benefiting patient prognosis, around one-third of UC patients exhibited an inadequate or limited response to IFX treatment. Therefore, we focused on identifying suitable indicators to predict response and prognosis to IFX therapy. We designated patients displaying clinical response following IFX induction therapy without the need for secondary replacement therapy as initial responders. The definition of clinical remission included that the partial Mayo scores decreasing at least 30% from initial assessment and the absolute rectal bleeding sub-scores being 0 or 1 (17). The nutritional status of patients may affect the efficacy of drug therapy, for example, Kurnool et al. (18) found obesity was a negative prognostic factor in patients on biologic therapy for UC (18), However we found no difference in BMI between initial responders and initial non-responders in this study [BMI, 21.70 (19.58, 23.67) VS 20.31 (18.60, 23.24), *P* = 0.154], This may be related to the range of BMI of the subjects in this study, so that the nutritional status of the patients (e.g., BMI) did not have significant effect on the outcome of the treatment. Among 157 UC patients, there were 89 responders and 68 non-responders after induction therapy. As shown in Figure 2, initial responders to IFX after the induction period exhibited lower baseline CAR [0.153 (0.033, 0.316)] and NAR [0.090 (0.062, 0.126)] compared to initial non-responders [CAR: 0.240 (0.096, 0.630), *P* = 0.013; NAR: 0.120 (0.083, 0.162), *P* = 0.020]. No notable distinctions between initial responders and initial non-responders were observed for CRP (*P* = 0.254), ALB (*P* = 0.091), and NEU (*P* = 0.567). In addition, we found that initial responders at the end of induction CAR [0.087 (0.028, 0.264] and NAR [0.079 (0.551, 0.100)] were decreased compared to baseline [CAR: 0.153 (0.033, 0.316), *P* = 0.906; NAR: 0.090 (0.062, 0.126), *P* = 0.083], and among initial non-responders, CAR [0.397 (0.197, 1.025)] and NAR [0.140 (0.101, 0.185)] at the end of induction were increased compared to baseline [CAR: 0.240 (0.096, 0.630), *P* = 0.961; NAR: 0.120 (0.083, 0.162), *P* = 0.065] (Figure 3). ROC curve analysis confirmed the predictive power of baseline CAR and NAR for IFX treatment response after the induction period. The optimum cut-off value for CAR was 0.509 (sensitivity 0.491, specificity 0.967, AUC = 0.758, 95% CI 0.670–0.846, *P* < 0.001), and the optimum cut-off value for NAR was 0.117 (sensitivity 0.518, specificity 0.727, AUC = 0.643, 95% CI 0.534–0.751, *P* = 0.017) (Figure 4), indicating significant differentiation between responders and non-responders following IFX induction treatment.

**FIGURE 5:**
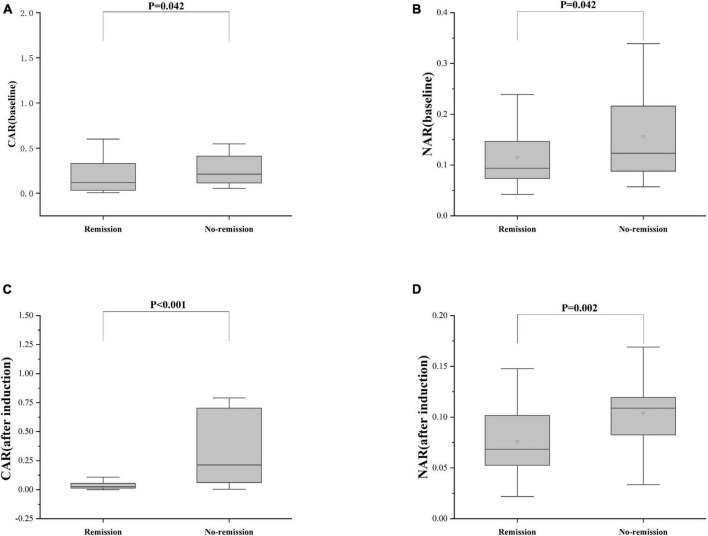
Differences of CAR and NAR between patients who achieve or do not achieve clinical remission. **(A)** CAR (baseline), the baseline CAR; **(B)** NAR (baseline), the baseline NAR; **(C)** CAR (after induction), CAR at the end of induction period; **(D)** NAR (after induction), NAR at the end of induction period. The comparison of two groups was performed by the Wilcoxon rank sum test [**(A)** CAR (baseline), *P* = 0.042; **(B)** NAR (baseline), *P* = 0.042; **(C)** CAR (after induction), *P* < 0.001; **(D)** NAR (after induction), *P* = 0.002].

**FIGURE 6:**
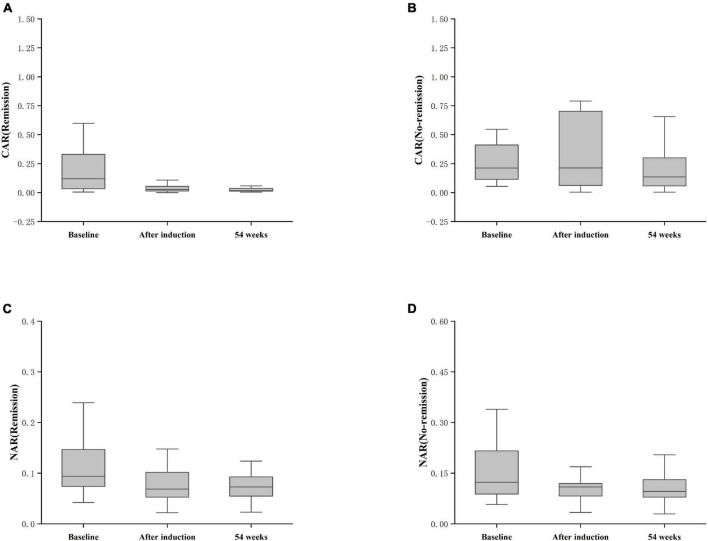
Differences in CAR and NAR at baseline, post-induction, and at 54 weeks between the remission and non-remission groups. **(A)** CAR (Remission), CAR achieving clinical remission at 54 weeks; **(B)** CAR (No-remission), CAR not achieving clinical remission at 54 weeks; **(C)** NAR (Remission), NAR achieving clinical remission at 54 weeks; **(D)** NAR (No-remission), NAR not achieving clinical remission at 54 weeks. The comparison was performed by the Kruskal-Wallis rank sum test.

**FIGURE 7:**
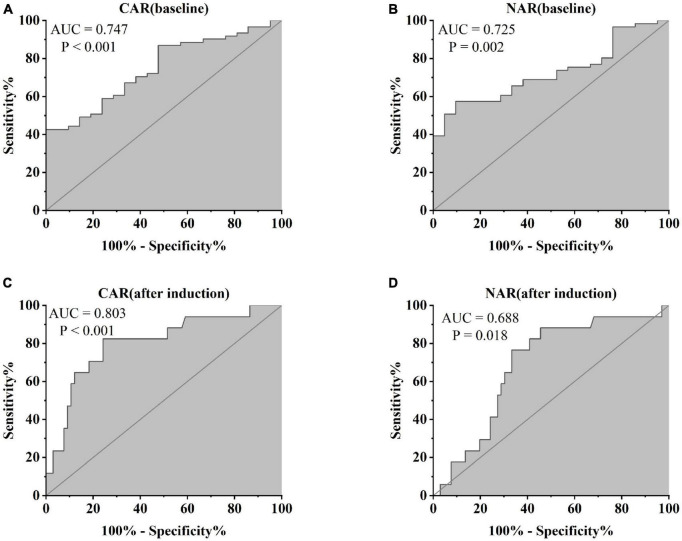
Abilities of **(A)** CAR (baseline), **(B)** NAR (baseline), **(C)** CAR (after induction), and **(D)** NAR (after induction) to predict clinical remission in UC patients after 54 weeks of IFX treatment [**(A)** CAR (baseline), AUC = 0.747, *P* < 0.001; **(B)** NAR (baseline) AUC = 0.725, *P* = 0.002; **(C)** CAR (after induction), AUC = 0.803, *P* < 0.001; **(D)** NAR (after induction), AUC = 0.688, *P* = 0.018].

Furthermore, patients failing to achieve clinical remission at 54 weeks exhibited higher baseline CAR [0.212 (0.114, 0.445)] and NAR [0.123 (0.088, 0.216)] compared to those who achieved clinical remission [CAR: 0.120 (0.032, 0.352), *P* = 0.042; NAR: 0.094 (0.073, 0.147), *P* = 0.042]. Similar distinctions were noted post-induction. Notably, patients achieving clinical remission demonstrated considerably lower CAR (after induction) [0.026 (0.014, 0.054)] compared to those not achieving remission [0.213 (0.061, 0.703), *P* < 0.001]. Additionally, patients achieving clinical remission exhibited significantly lower NAR (after induction) [0.068 (0.053, 0.112)] compared to those who did not attain remission [0.109 (0.083, 0.120), *P* = 0.002] (Figure 5). In addition, among patients achieving clinical remission, there were differences in the overall distribution of CAR and NAR in the three groups at baseline, after induction and at 54 weeks (CAR, *P* < 0.001; NAR, *P* < 0.001). Not only was the difference between after induction CAR and NAR and baseline CAR and NAR statistically significant (CAR, *P* < 0.001; NAR, *P* < 0.001), but the difference between baseline CAR and NAR and CAR and NAR at 54 weeks of therapy was also statistically significant (CAR, *P* < 0.001; NAR, *P* < 0.001). However, the difference between CAR and NAR after induction therapy and CAR and NAR at 54 weeks of therapy was not statistically significant (CAR, *P* = 0.887; NAR, *P* = 0.978). Nonetheless, among patients not achieving clinical remission, there were no statistical differences in the overall distribution of CAR and NAR among the three groups at baseline, after induction and at 54 weeks (CAR, *P* = 0.103; NAR, *P* = 0.102) (Table 3). As shown in Figure 6, we found that CAR and NAR in patients achieving clinical remission showed a downward trend over the treatment cycle, while CAR and NAR in those who not achieving clinical remission did not vary significantly. ROC curve analysis demonstrated the ability of CAR and NAR to predict clinical remission. The optimum cut-off value for baseline CAR was 0.423 (sensitivity 0.426, specificity approximately 1.000, AUC = 0.747, 95% CI 0.636–0.858, *P* < 0.001), and for baseline NAR was 0.120 (sensitivity 0.574, specificity 0.905, AUC = 0.725, 95% CI 0.616–0.835, *P* = 0.002). Simultaneously, the optimum cut-off value for CAR (after induction) was 0.061 (sensitivity 0.824, specificity 0.758, AUC = 0.803, 95% CI 0.680–0.925, *P* < 0.001), and for NAR (after induction) was 0.088 (sensitivity 0.765, specificity 0.667, AUC = 0.688, 95% CI 0.556–0.819, *P* = 0.018) (Figure 7). Hence, CAR and NAR could serve as predictive biomarkers for achieving clinical remission.

**TABLE 3:** Changes in CAR and NAR in the remission and non-remission groups.

	Baseline	After induction	54 weeks	*P*-value
CAR (Remission)	0.120 (0.032, 0.352)	0.026 (0.014, 0.054)*	0.017 (0.011, 0.037)*	<0.001
CAR (No-remission)	0.212 (0.114, 0.445)	0.213 (0.061, 0.703)	0.136 (0.057, 0.311)	0.103
NAR (Remission)	0.094 (0.074, 0.147)	0.068 (0.053, 0.102)*	0.073 (0.054, 0.094)*	<0.001
NAR (No-remission)	0.123 (0.088, 0.216)	0.109 (0.083, 0.120)	0.096 (0.079, 0.131)	0.102

The comparison was performed by the Kruskal-Wallis rank sum test. Comparisons between the two groups were made using Bonferroni at *P* < 0.05.

*Indicates statistically significant difference from baseline, there was no statistically significant difference between after induction and at 54 weeks.

## 4 Discussion

Ulcerative colitis remains a challenging condition among digestive diseases. Despite primarily affecting the intestinal mucosal layer, its persistent cycle of relapse and remission prolongs disease duration. Accurate monitoring of disease activity is pivotal for selecting optimal treatment strategies. While endoscopy is the current gold standard to assess UC activity (19), its invasive nature can exacerbate the condition, and some patients exhibit poor compliance. Therefore, we urgently need to search for appropriate biomarkers with the characteristics of being non-invasive, easy to implement, and having high sensitivity and specificity (20). However, to date, no identified biomarker has met all these requirements. Previous studies have highlighted the anti-Saccharomyces cerevisiae antibody (ASCA) as a reliable biomarker for CD, yet its prevalence in celiac disease has limited its exclusive use (21–23). The identification of suitable biomarkers could not only minimize the invasiveness associated with endoscopy but also alleviate the medical burden on patients. The urgent need for new biomarkers is crucial to manage UC treatment. Accurate assessment of disease activity is imperative in devising optimal treatment strategies and enhancing the prognosis for UC patients.

In the present investigation, we investigated variations in two biomarkers in UC patients, CAR and NAR. Firstly, both CAR and NAR exhibited significant elevation in UC patients compared with healthy controls. Secondly, we observed the positive correlation between CAR, NAR, and the modified Mayo score. Thirdly, CAR and NAR demonstrated potential predictive capabilities to distinguish responders to IFX induction therapy from primary non-responders among UC patients. Fourthly, our study revealed CAR and NAR levels could predict the likelihood of achieving clinical remission at 54 weeks of IFX therapy. We aimed to ascertain whether CAR and NAR could serve as suitable indicators for the diagnosis and treatment of UC patients.

C-reactive protein-to-albumin ratio was used as an indicator reflecting the balance between inflammation and nutritional status. Recent literature has extensively discussed CAR and its relevance to various diseases. For instance, Wu et al. (24) in a meta-analysis revealed that decreased CAR levels could predict improved overall survival in cancer patients. Additionally, Kim et al. (25) reported prognostic value of CAR in predicting mortality among patients with septic shock. Qin et al. (26) demonstrated that CAR was used to assess CD activity. Liu et al. (27) illustrated the positive correlation between higher CAR levels and greater disease activity. We observed the positive association between CAR and the modified Mayo score in this investigation, signifying the utility of CAR as an indicator for assessing UC activity, which was consistent with previous research. Furthermore, CAR displayed the superior predictive value for IFX treatment response compared to CRP and ALB. Notably, CAR exhibited the ability to predict clinical remission in UC patients undergoing 54 weeks of IFX therapy, a crucial factor impacting disease prognosis.

Recent studies have explored numerous biomarkers for evaluating disease activity. Neutrophils, crucial phagocytes responsible for acute inflammatory responses and key mediators of the immune system, were one of the most common biomarkers to assess disease activity (28). Meanwhile, NAR has emerged as a novel systemic indicator of inflammation, extensively utilized in evaluating inflammation, vascular disease, and cancer (24, 29). Additionally, studies have revealed lowered serum albumin (ALB) levels in IBD patients, potentially affecting the therapeutic efficacy of biologic agents (25). Notably, research by Inflammatory Bowel Disease Group et al. (9) highlighted the heightened utility of NAR in reflecting UC activity and systemic inflammatory burden. However, existing research on NAR predicting response to IFX therapy in IBD patients was limited. Our investigation unveiled that NAR not only served as a valuable biomarker to diagnose diseases and assess disease activity but also demonstrated predictive potential in discerning between UC patients who respond or do not respond to IFX induction treatment. Furthermore, NAR exhibited the capability to differentiate between clinical remission and non-clinical remission among UC patients undergoing 54 weeks of IFX therapy, offering valuable guidance in selecting treatment strategies.

Biologics have assumed the significant role in UC treatment, with IFX, a common anti-TNF drug, notably contributing to clinical remission and mucosal healing in UC patients. However, not all individuals derive equal benefit from IFX treatment; approximately one-third of patients exhibit unresponsiveness following IFX induction therapy, known as “primary non-responsive” (30). Therefore, predicting response to IFX induction therapy in UC patients remains crucial. Previous studies explored predictors for IFX treatment response, including disease course, CRP, and ALB, yet results have been inconclusive. Some studies have established the correlation between the response rate to IFX and serum albumin levels in UC patients, demonstrating that lower blood albumin levels were related with poorer response rates (30, 31). Furthermore, some investigations revealed the correlation between IFX serum levels and serum albumin levels, indicating that lower serum albumin levels corresponded to decreased IFX serum levels. However, the predictive role of ALB and IFX serum levels in determining IFX response among UC patients remains uncertain. In our study, we found that CAR and NAR may be effective tools for prognosticating response to IFX induction therapy and clinical remission after 54 weeks of IFX therapy. If CAR and NAR were implemented in clinical practice, they could significantly reduce the frequency of endoscopic procedures and alleviate the medical burden on UC patients.

In conclusion, our study underscored the significance of two biomarkers, CAR and NAR, as sensitive indicators for assessing disease activity in UC patients. Importantly, we have demonstrated their dual capacity not only to predict response to IFX induction therapy in UC patients but also to forecast the potential achievement of clinical remission following 54 weeks of IFX therapy. However, this research has limitations. Firstly, due to retrospective study, retrospective bias may exist, and the paper relied on a small sample size, which highlighted the need for larger-scale investigations to validate our findings. Secondly, the applicability of these predictors to other biological agents like Vedolizumab and Ustekinumab remains unconfirmed. Furthermore, we failed to measure IFX serum levels and antibodies, thus requiring further exploration of the relationship between IFX trough concentrations or IFX autoantibodies with CAR and NAR. Nevertheless, our results offer compelling evidence supporting the potential application of CAR and NAR in diagnosis, monitoring disease activity, predicting IFX response, and forecasting clinical remission in UC patients.

## Data availability statement

The original contributions presented in this study are included in this article/supplementary material, further inquiries can be directed to the corresponding author.

## Ethics statement

The studies involving humans were approved by the Ethics Review Board of the Clinical Research Institution of the First Affiliated Hospital of Zhengzhou University. The studies were conducted in accordance with the local legislation and institutional requirements. Written informed consent for participation was not required from the participants or the participants’ legal guardians/next of kin in accordance with the national legislation and institutional requirements.

## Author contributions

YZ: Writing – review and editing, Writing – original draft, Methodology, Data curation, Conceptualization. FX: Writing – review and editing, Supervision, Methodology, Conceptualization. YL: Writing – review and editing, Methodology, Data curation. BC: Writing – review and editing, Data curation.

## References

[1] Berre C Honap S Peyrin-Biroulet L. Ulcerative colitis. *Lancet.* (2023) 402:571–84. 37573077 10.1016/S0140-6736(23)00966-2

[2] Navaneethan U Parasa S Venkatesh P Trikudanathan G Shen B. Prevalence and risk factors for colonic perforation during colonoscopy in hospitalized inflammatory bowel disease patients. *J Crohns Colitis.* (2011) 5:189–95. 21575880 10.1016/j.crohns.2010.12.005

[3] Buisson A Gonzalez F Poullenot F Nancey S Sollellis E Fumery M Comparative acceptability and perceived clinical utility of monitoring tools: A nationwide survey of patients with inflammatory bowel disease. *Inflamm Bowel Dis.* (2017) 23:1425–33. 10.1097/MIB.0000000000001140 28570431

[4] Abej E El-Matary W Singh H Bernstein C. The utility of fecal calprotectin in the real-world clinical care of patients with inflammatory bowel disease. *Can J Gastroenterol Hepatol.* (2016) 2016:2483261. 10.1155/2016/2483261 PMC5059522 27774443

[5] Sands B. Biomarkers of inflammation in inflammatory bowel disease. *Gastroenterology.* (2015) 149:1275–85. 26166315 10.1053/j.gastro.2015.07.003

[6] Zhou Z Zhang Y Yang X Pan Y Li P Gao C Clinical significance of novel neutrophil-based biomarkers in the diagnosis and prediction of response to infliximab therapy in Crohn’s disease. *Front Immunol.* (2022) 13:865968. 10.3389/fimmu.2022.865968 35309310 PMC8931310

[7] Torun S Tunc B Suvak B Yildiz H Tas A Sayilir A Assessment of neutrophil-lymphocyte ratio in ulcerative colitis: A promising marker in predicting disease severity. *Clin Res Hepatol Gastroenterol.* (2012) 36:491–7. 10.1016/j.clinre.2012.06.004 22841412

[8] Bertani L Rossari F Barberio B Demarzo M Tapete G Albano E Novel prognostic biomarkers of mucosal healing in ulcerative colitis patients treated with anti-TNF: Neutrophil-to-lymphocyte ratio and platelet-to-lymphocyte ratio. *Inflamm Bowel Dis.* (2020) 10:1579–87. 10.1093/ibd/izaa062 32232392

[9] Inflammatory Bowel Disease Group, Chinese Society of Gastroenterology, Chinese Medical Association. Chinese consensus on diagnosis and treatment of inflammatory bowel disease (Beijing, 2018). *Chin J Pract Intern Med.* (2018) 38:796–813.

[10] Zhao X Li L Li X Li J Wang D Zhang H. The relationship between serum bilirubin and inflammatory bowel disease. *Mediat Inflamm.* (2019) 2019:5256460. 10.1155/2019/5256460 PMC6501121 31148945

[11] Su Q Li X Mo W Yang Z. Low serum bilirubin, albumin, and uric acid levels in patients with Crohn’s disease. *Medicine (Baltimore).* (2019) 98:e15664. 10.1097/MD.0000000000015664 31083269 PMC6531115

[12] Ye Z Yang Y. Current status and progress of serum C-reactive protein, albumin and its ratio in inflammatory related diseases. *Med Recapitulate.* (2017) 23:3979–83.

[13] Simundic A. Measures of diagnostic accuracy: Basic definitions. *EJIFCC.* (2009) 19:203–11. 27683318 PMC4975285

[14] Lewis J Chuai S Nessel L Lichtenstein G Aberra F Ellenberg J. Use of the noninvasive components of the Mayo score to assess clinical response in ulcerative colitis. *Inflamm Bowel Dis.* (2008) 14:1660–6. 10.1002/ibd.20520 18623174 PMC2597552

[15] Stabroth-Akil D Leifeld L Pfützer R Morgenstern J Kruis W. The effect of body weight on the severity and clinical course of ulcerative colitis. *Int J Colorectal Dis.* (2015) 30:237–42. 25392256 10.1007/s00384-014-2051-3

[16] Yerushalmy-Feler A Ben-Tov A Weintraub Y Amir A Galai T Moran-Lev H High and low body mass index may predict severe disease course in children with inflammatory bowel disease. *Scand J Gastroenterol.* (2018) 53:708–13. 10.1080/00365521.2018.1464595 29688090

[17] Armuzzi A Pugliese D Danese S Rizzo G Felice C Marzo M Infliximab in steroid-dependent ulcerative colitis: Effectiveness and predictors of clinical and endoscopic remission. *Inflamm Bowel Dis.* (2013) 19:1065–72. 23448790 10.1097/MIB.0b013e3182802909

[18] Kurnool S Nguyen N Proudfoot J Dulai P Boland B Vande Casteele N High body mass index is associated with increased risk of treatment failure and surgery in biologic-treated patients with ulcerative colitis. *Aliment Pharmacol Ther.* (2018) 47:1472–9. 10.1111/apt.14665 29665045 PMC5992082

[19] Sandborn W Colombel J D’Haens G Van Assche G Wolf D Kron M One-year maintenance outcomes among patients with moderately-to-severely active ulcerative colitis who responded to induction therapy with adalimumab: Subgroup analyses from ULTRA 2. *Aliment Pharmacol Ther.* (2013) 37:204–13. 10.1111/apt.12145 23173821

[20] Bouguen G Levesque B Pola S Evans E Sandborn W. Endoscopic assessment and treating to target increase the likelihood of mucosal healing in patients with Crohn’s disease. *Clin Gastroenterol Hepatol.* (2014) 12:978–85. 24246770 10.1016/j.cgh.2013.11.005

[21] Vermeire S Van Assche G Rutgeerts P. Laboratory markers in IBD: Useful, magic, or unnecessary toys? *Gut.* (2006) 55:426–31. 16474109 10.1136/gut.2005.069476 PMC1856093

[22] Vermeire S Peeters M Vlietinck R Joossens S Den Hond E Bulteel V Anti-saccharomyces cerevisiae antibodies (ASCA), phenotypes of IBD, and intestinal permeability: A study in IBD families. *Inflamm Bowel Dis.* (2001) 7:8–15. 10.1097/00054725-200102000-00002 11233666

[23] Granito A Zauli D Muratori P Muratori L Grassi A Bortolotti R Anti-saccharomyces cerevisiae and perinuclear anti-neutrophil cytoplasmic antibodies in coeliac disease before and after gluten-free diet. *Aliment Pharmacol Ther.* (2005) 21:881–7. 10.1111/j.1365-2036.2005.02417.x 15801923

[24] Wu J Tan W Chen L Huang Z Mai S. Clinicopathologic and prognostic significance of C-reactive protein/albumin ratio in patients with solid tumors: An updated systemic review and meta-analysis. *Oncotarget.* (2018) 9:13934–47. 10.18632/oncotarget.24172 29568406 PMC5862627

[25] Kim M Ahn J Song J Choi H Ann HW Kim JK The C-reactive protein/albumin ratio as an independent predictor of mortality in patients with severe sepsis or septic shock treated with early goal-directed therapy. *PLoS One.* (2015) 10:e0132109. 10.1371/journal.pone.0132109 26158725 PMC4497596

[26] Qin G Tu J Liu L Luo L Wu J Tao L Serum albumin and C-reactive protein/albumin ratio are useful biomarkers of Crohn’s disease activity. *Med Sci Monit.* (2016) 22:4393–400. 10.12659/msm.897460 27848931 PMC12574461

[27] Liu A Lv H Tan B Shu H Yang H Li J Accuracy of the highly sensitive C-reactive protein/albumin ratio to determine disease activity in inflammatory Bowel disease. *Medicine.* (2021) 100:25200. 10.1097/MD.0000000000025200 33832080 PMC8036110

[28] Nishida Y Hosomi S Yamagami H Yukawa T Otani K Nagami Y Neutrophil-to-lymphocyte ratio for predicting loss of response to infliximab in ulcerative colitis. *PLoS One.* (2017) 12:e0169845. 10.1371/journal.pone.0169845 28076386 PMC5226844

[29] Varim C Yaylaci S Demirci T Kaya T Nalbant A Dheir H Neutrophil count to albumin ratio as a new predictor of mortality in patients with COVID-19 infection. *Rev Assoc Med Bras.* (2020) 66:77–81. 10.1590/1806-9282.66.S2.77 32965361

[30] Steenholdt C Bendtzen K Brynskov J Thomsen O Munck L Christensen L Changes in serum trough levels of infliximab during treatment intensification but not in anti-infliximab antibody detection are associated with clinical outcomes after therapeutic failure in Crohn’s disease. *J Crohns Colitis.* (2015) 9:238–45. 10.1093/ecco-jcc/jjv004 25576753

[31] Arias M Vande Casteele N Vermeire S de Buck van Overstraeten A Billiet T Baert F A panel to predict long-term outcome of infliximab therapy for patients with ulcerative colitis. *Clin Gastroenterol Hepatol.* (2015) 13:531–8. 10.1016/j.cgh.2014.07.055 25117777

